# Prospective Associations Between Preschool Exposure to Violent Televiewing and Externalizing Behavior in Middle Adolescent Boys and Girls

**DOI:** 10.3390/ijerph22010129

**Published:** 2025-01-20

**Authors:** Linda S. Pagani, Amélie Gilker Beauchamp, Laurie-Anne Kosak, Kianoush Harandian, Claudio Longobardi, Eric Dubow

**Affiliations:** 1 School of Psycho-Education, Université de Montréal, Montreal, QC H3C 3J7, Canada; amelie.gilker.beauchamp@umontreal.ca (A.G.B.); laurie-anne.kosak@umontreal.ca (L.-A.K.); kianoush.harandian@umontreal.ca (K.H.); 2 Sainte-Justine’s Pediatric Hospital Research Center, Université de Montréal, Montreal, QC H3C 3J7, Canada; 3 School Environment Research Group, Université de Montréal, Montreal, QC H3C 3J7, Canada; 4 Department of Psychology, Università degli Studi di Torino, 20126 Turin, Italy; claudio.longobardi@unito.it; 5 Department of Psychology, Bowling Green State University, Bowling Green, OH 43403, USA; edubow@bgsu.edu; 6 Institute for Social Research, The University of Michigan, Ann Arbor, MI 48104, USA

**Keywords:** preschool development, psycho-social risks, screen violence, youth, maladaptive development, aggression

## Abstract

Objective. Early childhood exposure to violent media content represents an actionable target for preventive intervention. The associated risks for later aggressive behavior have been established in childhood, but few studies have explored widespread long-term associations with antisocial behavior. We investigate prospective associations between exposure to violent television content in early childhood and subsequent antisocial behavior in mid-adolescence. Method. Participants are 963 girls and 982 boys from the Quebec Longitudinal Study of Child Development (QLSCD) birth cohort. Parents reported the frequency of their child’s exposure to violent television content at ages 3.5 and 4.5 years. Four indicators of antisocial behavior were self-reported by participants at age 15 years. These indicators were linearly regressed on exposure to violent television content at ages 3.5 and 4.5 years. All analyses, stratified by sex, controlled for pre-existing and concurrent potential individual and family confounding variables. Results. For boys, preschool violent televiewing was associated with increases in proactive aggression (β = 0.065; 95% CI, 0.001 to 0.089), physical aggression (β = 0.074; 95% CI, 0.040 to 0.487), and antisocial behavior (β = 0.076; 95% CI, 0.013 to 0.140) by mid-adolescence. No prospective associations were found for girls. Conclusions. This study of typically developing children demonstrates long-term perils associated with early exposure to violent content in childhood. We observed risks for aggressive and delinquent behavior in boys, more than a decade later. Preventive intervention campaigns that target knowledge transfer to parents and communities regarding the potential insidious consequences of preschool exposure promise more optimal development in youth.

## 1. Introduction

Young children are often directly or indirectly exposed to the omnipresence of overt and covert violence throughout most media [1]. Research suggests that exposure to violent content has both short- and long-term negative risks for child development [2,3,4,5]. Given the increasingly varied use of technology in our daily lives, across contexts and devices, the associated perils of violent content are difficult to examine at the present time [6]. Using historical data at a time when television screens were the main exposure offers its methodological advantages of limiting competing ubiquitous technology, as seen in earlier seminal research.

Violent media is characterized by physical aggression, verbal aggression, and relational aggression and refers to any visual representation from digital media depicting situations that intentionally attempt or cause harm to others [7]. Children are attracted to fast-paced, stimulating violent content, which often features appealing characters like superheroes who commit and are rewarded for aggressive acts, thus increasing the likelihood of exposure [8]. 

In an experimental study, one group would be exposed to highly violent content while another would not. However, ethical constraints prevent this among very young children. Non-experimental studies assess exposure to violence in natural settings without altering it. Like a natural experiment, correlational studies can test real-life experiences that ethically would be difficult to investigate in preschoolers, for example. The long-term psycho-social risks associated with early exposure remain the object of debate.

On one hand, some researchers, such as Ferguson (2015) have not found a connection between television violence and violent behavior [9]. However, not achieving a significant effect and thus failing to reject the null hypothesis does not necessarily imply that there is no specific effect. What is more, if an effect exists, it might be small or particular to an individual susceptibility characteristic (like sex or vulnerable developmental periods) or to environmental influences in microsystems such as the family or household [10,11].

On the other hand, there are compelling investigations that show otherwise [1,2,3,4,5,6,7,8,9]. Early violent televiewing correlates with increased short- and long-term externalizing behaviors. A study of American school-aged children into their mid-twenties found the highest exposed 20% showed significantly more aggression 15 years later [12]. For males, this meant more physical violence, while for females, this meant more indirect violence. Specifically, males exposed to much violence as children were twice as likely to assault partners and 18% more likely to threaten with dangerous objects. Using a historical birth cohort born between 1972 and 1973 in New Zealand, Robertson et al. (2013) also found that violent televiewing in childhood also links to higher antisocial traits and early adult criminal convictions [13]. This was above and beyond confounding variables such as early antisocial behavior, childhood IQ, socioeconomic status, and parental control.

These long-term associations are supported by a multi-theorical backdrop. Bandura’s observational learning theory suggests how early exposure to violent media can be a risk factor for children. In his bobo doll experiment, he found that behavior was learned through observing models. The effect of vicarious learning was particularly salient when the child, of average age 4 years and 4 months, identified with the perpetrator and when the behavior seemed to be rewarded [14]. Consequently, during repeated learning, young children may imitate violent actions seen on screen, as consequences are seldom part of the storyline, and characters are often encouraged and rewarded to act aggressively. Repeated violent television exposure, as a typical childhood experience without adult intervention, can downplay the consequences of aggression as acceptable or rewarding on some relational level [8,15,16]. 

The General Aggression Model suggests that social, environmental, and personal factors interact to shape individual aggression [17]. This has implications for cognitively and socially inexperienced minds who are vicariously exposed to antisocial behavior in early childhood. Repeated contact with violence can affect perceptions of social situations, as well as personality characteristics, and thus alter relationships and interactions with others. Aggressive beliefs, expectations, and scripts can contribute to the development of an aggressive personality [18,19,20]. Through desensitization, driven by a habituation process, whereby individual emotional reactions to a stimulus diminish over time due to repeated exposure to violence, children may eventually trivialize aggression and view it as an acceptable process in resolving social conflicts or competition [21]. This is because, by being overexposed to violence in the media, emotional reactions to the sight of violence diminish over time, as the developing mind acculturates to such content. Desensitization may reduce empathy toward violence victims and decrease willingness to assist others in real-life situations [17].

The extant research on the relationship between preschool exposure to media violence and psycho-social outcomes in adolescence has faced various limitations. Most longitudinal studies on this topic start during school age, overlooking the preschool years. However, experiences during the early years of life represent a critical period for children’s subsequent cognitive, physical, and socio-emotional development [22]. Shaping the child’s future psycho-social trajectory, healthy developmental conditions are crucial for the child to grow into an independent individual who can thrive and respect societal norms [23]. 

Finally, past research has mostly treated sex as a covariate (e.g., [24]). This ignores the established diversity in both biological and social influences. Males and females face distinct attitudes, norms, and education, affecting their media exposure and activities [25]. Boys are more inclined toward action/fast-paced media and direct aggression and girls are more attracted to more relational/emotional content and indirect aggression [26]. Thus, given that males and females typically experience risk and protective factors differently, due to biological and social expectations, treating boys and girls as distinct entities in analysis becomes essential to advance knowledge in the field [27].

The purpose of this study is to investigate long-term psycho-social risks linked with violent televiewing during preschool years. Specifically, the study uses a birth cohort of girls and boys to examine prospective associations between exposure to violent media at ages 3.5 and 4.5 years and antisocial behavior at age 15 years. Data are from multiple sources and include parents, trained examiners, and self-report. It is expected that exposure to violent television content during the preschool years will prospectively forecast antisocial behavior by mid-adolescence, above and beyond pre-existing and concurrent individual and family confounding characteristics.

## 2. Methods

### 2.1. Participants

The participants of this study are a birth cohort from the Quebec Longitudinal Study of Child Development (QLSCD), conducted by the *Institut de la Statistique du Quebec*, with the aim of studying factors influencing the social adaptation and academic success of millennial children from the province of Quebec, Canada (http://www.iamillbe.stat.gouv.qc.ca/default_an.htm, accessed on 12 December 2024). Initially, the QLSCD randomly selected a stratified sample of 2837 infants born between 1997 and 1998 in the province of Quebec, from the provincial birth register. Ultimately, 2223 children and their families were included, representing 82% of the initial population, as among the initially selected families, 93 were deemed ineligible, 172 could not be traced, 14 could not be reached, and 438 refused to participate. In the first phase (1998–2002), children were followed annually from the age of 5 months and then were followed twice a year upon entering elementary school during the second phase (2003–2010). In the third phase (2011–2015), starting from the age of 13, the participants were followed every two years until they reached adulthood. Informed consent was obtained from parents in every phase of data collection and by children from the school-age phases. In this study, 1945 preschool children (50.4% boys) with complete data on exposure to violent television content at age 3.5 or 4.5 years were examined (2001–2002). Complete data for outcome variables at age 15 years (2013) were available for 72.27% of the sample.

### 2.2. Measures: Predictor (Violent Televiewing at Ages 3.5 and 4.5 Years)

At ages of 3.5 and 4.5 years, the person who is most familiar with the child (the mother in 98% of cases) answered the question “How often does your child watch television shows or movies that have a lot of violence in them?”. This item was developed for the Quebec Longitudinal Study of Child Development by the *Institut de la Statistique du Québec*. Responses were reported on a Likert scale ranging between 0 (never), 1 (rarely), 2 (sometimes), and 3 (often). An average score was calculated for each child.

### 2.3. Measures: Outcomes (Externalizing Behavior at Age 15 Years)

To measure externalizing behavior at the age of 15 years, participants completed online the Mental Health and Maladjustment in Adolescence questionnaire [28]. This questionnaire aims to assess the mental health of the youth. It also aims to better understand the contribution of risk and protective factors during childhood and adolescence. Four outcomes were built by summing the corresponding items for each of the following scales. Responses were coded on a 3-point Likert scale ranging between 0 (never true), 1 (sometimes true), and 2 (often true). For all measures, higher values indicate more externalizing behavior [4,6,28].

*Proactive aggression* was measured using a scale of 4 items: “In the past 12 months, I threatened to hit someone to get what I wanted/ I hit someone who had done nothing/ I threatened to beat someone to make them do something they didn’t want to do/ I threatened to hit someone in order to steal from them”. The values for this scale ranged from 0 to 8 (a = 0.78).

*Reactive aggression* was measured using a scale of 3 items: “In the past 12 months, I hit someone who hurt me even if it wasn’t on purpose/ I hit someone who threatened me/ when I was bumped, I hit the person who bumped me even if it wasn’t on purpose”. The values for this scale ranged from 0 to 6 (a = 0.63).

*Physical aggression* was measured using a scale of 17 items: “In the past 12 months, I participated in gang fights/ I threatened to hit someone to get what I wanted/ I hit someone who hurt me even if it wasn’t on purpose/ I hit someone who threatened me/ I said hurtful things behind someone’s back/ I engaged in cyberbullying (insults, threats, intimidation, etc.) on the Internet or via cellphone towards another youth/ I yelled names, insulted, or said hurtful things to others/ I intentionally hurt someone to the point where they needed medical attention/ when I was bumped, I hit the person who bumped me even if it wasn’t on purpose/ I used a weapon (e.g., stick, stone, knife) to fight with someone/ I beat someone who hadn’t done anything to me/ I mocked or laughed at someone/ I prevented someone from joining my group when they wanted to/ I struggled with the idea of seriously hurting someone/ I threatened to beat someone to force them to do something they didn’t want to do/ I threatened to hit someone in order to steal from them/ I ‘taxed’ another youth”. The values for this scale ranged from 0 to 32 (a = 0.88).

*Antisocial behavior* was measured using a scale of 5 items: “In the past 12 months, I appeared before a judge for doing something wrong/ I was placed in a Youth Center for doing something wrong/ I was convicted for doing something wrong/ I was arrested by the police for doing something wrong/ I was questioned by police about something they thought I had done”. The values for this scale ranged from 0 to 10 (a = 0.67).

### 2.4. Measures: Individual and Family Control Variables (Between Ages 5 Months and 15 Years)

Pre-existing and concurrent child and family characteristics were selected based on the theoretical and empirical literature on TV violence effects among youth. Responses indicating a risk were coded as 1, and those indicating no risk were coded as 0. The following characteristics were reported by the person who was most familiar with the child. Data on sex were obtained at age 5 months (1 = boy) from the Labour Force Survey [29]. Perception of difficult temperament at 1.5 years (1 = 1 SD above the mean) was measured by the Infant Characteristics Questionnaire [30] (a = 0.80). Baseline physical aggression at age 1.5 years (1 = 1 SD above the mean) and exposure to physical aggression in the family home at age 3.5 years (1 = some exposure) were measured by the National Longitudinal Study on Children and Youth (NLSCY) [4,6,28] (a = 0.80). To measure neurocognitive abilities at age 2.5 years (1 = 1 SD above the mean), the Object Placement Imitation task was administered to the child by the examiner [31]. Total screen time at age 15 years, including time spent using the computer, video games, and television (1 = 1 SD above the mean) was self-reported by the youth [6].

Maternal depressive symptoms at age 5 months (1 = 1 SD above the mean) were measured by the Center for Epidemiological Studies Depression Scale [32] (a = 0.81). Maternal education at age 5 months (1 = did not finish high school) was measured by the General Social Survey on Work and Education [33]. Parental history of antisocial behaviors at age 5 months (1 = 1 SD above the mean) was measured by the National Institute of Mental Health-Diagnostic Interview Schedule [34] (a = 0.62). Family dysfunction at age 1.5 years (1 = 1 SD above the mean) was measured by the McMaster Family Assessment Device [35] (a = 0.84). Hostile parenting practices by the mother were measured by the interviewer at age 1.5 years (1 = 1 SD above the mean) using the Home Observation for Measurement of the Environment—Infant Version [21] (a = 0.77). The income sufficiency (1 = insufficient income) was measured at age 3.5 years according to the Low-Income Measure (LIM) by the Survey on Labor and Income Dynamics [36]. Family configuration at age 15 years (1 = single parent) is a measure from the NLSCY [29]. The questionnaires used were carefully selected by the QLSCD research team based on their validity and relevance. The sources and justifications for the questions, scales, forms, and tests used can be retrieved at the following link: https://www.jesuisjeserai.stat.gouv.qc.ca/, accessed on 12 December 2024.

### 2.5. Data Analytic Strategies

Long-term prospective associations were estimated using the ordinary least squares multiple regression. Four regressions were computed, one for each measure of externalizing behavior. Externalizing behavior at age 15 years was linearly regressed on exposure to violent television content between ages 3.5 and 4.5 years. To mitigate potential bias in the findings, the model incorporated pre-existing and concurrent child and family characteristics, which are known or suspected to influence the explanation of the relationship between the predictor and the outcome. For each regression, the predictor and control variables were introduced simultaneously. The analyses were stratified by sex, treating boys and girls as two distinct populations.

As expected in a longitudinal study, some data were missing across participants. To correct potential or actual attrition bias, we conducted multiple imputation procedures with SPSS v.26 software. In total, 20 datasets were estimated and aggregated before conducting the analyses, to create a reliable estimate of missing data variance.

### 2.6. Between-Group Differences for Complete and Incomplete Data

This longitudinal study contained incomplete data that were analyzed using chi-squared tests. Boys with complete data were more likely to have had mothers with a high school diploma (χ^2^ (1, N = 979) = 5.587, *p* < 0.05), had less depressed mothers (χ^2^ (1, N = 982) = 6.918, *p* < 0.01), lived in sufficient income households (χ^2^ (1, N = 942) = 4.676, *p* < 0.05), had better neurocognitive abilities (1, N = 982) = 5.990, *p* < 0.05), and had less total screen time (χ^2^ (1, N = 982) = 50.669, *p* < 0.001) compared to boys with incomplete data. Girls with complete data lived in sufficient income households (χ^2^ (1, N = 963) = 6.440, *p* < 0.05), were exposed to less aggressivity at home (χ^2^ (1, N = 963) = 4.852, *p* < 0.05), lived with two parents (χ^2^ (1, N = 963) = 53.789, *p* < 0.001), and had less total screen time (χ^2^ (1, N = 963) = 41.559, *p* < 0.001) compared to girls with incomplete data.

## 3. Results

Table 1 reports descriptive statistics for the predictor, outcome, and control variables. Between ages 3.5 and 4.5 years, most girls had never been exposed to violent media and the majority of boys had been exposed to violent media at various frequencies.

Table 2 documents unstandardized regression coefficients (standard error) which reflect the relationship between pre-existing and concurrent child and family characteristics and violent televiewing. For boys, an increase in exposure to physical aggression at home at age 3.5 years (β = 0.098, *p* = 0.002, 95% confidence interval [CI], 0.119–0.534), in parental history of antisocial behavior at age 5 months (β = 0.103, *p* = 0.001, 95% confidence interval [CI], 0.088–0.355), as well as in baseline aggressive behaviors at age 1.5 year (β = 0.077, *p* = 0.025, 95% confidence interval [CI], 0.022–0.336) were associated with exposure to violent television content. For girls, an increase in exposure to physical aggression at home at age 3.5 years (β = 0.100, *p* = 0.002, 95% confidence interval [CI], 0.108–0.483), in parental history of antisocial behavior at age 5 months (β = 0.081, *p* = 0.012, 95% confidence interval [CI], 0.035–0.282), in low family income at age 3,5 years (β = 0.073, *p* = 0.034, 95% confidence interval [CI], 0.010–0.269), in baseline aggressive behavior at age 1.5 years (β = 0.088, *p* = 0.007, 95% confidence interval [CI], 0.046–0.294), and in overall screen time at age 15 years (β = 0.073, *p* = 0.022, 95% confidence interval [CI], 0.022–0.286) were associated with exposure to violent television content.

Table 3 reports unstandardized regression coefficients (standard error) that reflect the relationship between preschool exposure to violent televiewing and externalizing behavior at age 15 years for girls and boys. For boys only, results indicated risks associated with violent television exposure between ages 3.5 and 4.5 years, controlling for all early and concurrent covariates. The following results are presented using standardized regression coefficients.

Preschool violent televiewing in boys predicted higher levels of self-reported proactive aggression, physical aggression, and antisocial behavior in adolescence. Compared with boys who experienced less television violence exposure, preschool exposure to violent content predicted a 6,5% unit increase in proactive aggression (*p* = 0.043, 95% confidence interval [CI], 0.001–0.089), a 7,4% unit increase in physical aggression (*p* = 0.021, 95% confidence interval [CI], 0.040–0.487) as well as a 7,6% unit increase in antisocial behavior (*p* = 0.013, 95% confidence interval [CI], 0.013–0.140) at age 15 years. In terms of the covariates, overall screen time, parental antisocial behavior, maternal depressive symptoms, maternal education, family income, and family dysfunction were associated with indicators of externalizing behavior.

Preschool violent televiewing in girls does not predict higher levels of self-reported indicators of externalizing behavior in adolescence. In terms of the covariates, physical aggression exposure at home, parental antisocial behavior, overall screen time, family income, and family configuration were associated with indicators of externalizing behavior.

## 4. Discussion

In the past decade, a task force of experts from the American Psychological Association (APA) critically examined and meta-analyzed the existing literature on violent video game exposure from 2009 to 2013 [37]. They concluded that exposure was associated with increased physiological arousal and aggressive behavior, cognitions, and affect. They also found compelling associations with desensitization and reduced empathy. Effect sizes were consistent with prior meta-analytic findings conducted between 2005 and 2013, suggesting stable evidence and underscoring male vulnerability over time. However, this technical report had insufficient evidence for a connection between violence exposure and delinquency or criminal behavior. As expected, our biostatistical/epidemiological design, which used multiple data resources at different points in child development with a low-risk middle-class population forecasted net linear prospective associations between parent-reported preschool exposure to violent content and subsequent self-reported externalizing outcomes, 11 years later.

This study specifically documents long-term associated risks of self-reported physical and proactive aggression in adolescent males. Being exposed to violent content in early childhood predicted later aggressive behaviors such as hitting or beating another person, with the intention of obtaining something or stealing, with or without any apparent reason. These risks include threats, insults, and involvement in gang fights. The use of weapons is also among the behavioral outcomes predicted by exposure to childhood television violence in this study. Our findings support previous concerns about the insidious perils of viewing violence in early childhood for later developmental pathology [1,2,37].

Most importantly, we found long-term risks associated with self-reported antisocial behavior in boys. The viewing of violent content in early childhood predicted, in 15-year-old adolescents, appearances in court for offenses, placements in youth centers, and interactions with law enforcement. Self-reported violent tendencies at age 15 years do not augur well for subsequent development, because such an inclination tends to persist and influence difficulties in personal, family, and school spheres. Aggressive adolescent males specifically exhibit more long-term depressive symptoms and stress, lower self-esteem, reduced empathy, and lower life satisfaction in adulthood. They also have greater chances of less effective communication skills and lower cohesion with their family, even years after adolescence [3,38].

One should not underestimate the developmental impact of a small significant effect, as it can snowball over time, because this effect can influence behavioral choices (values in action) over the life course. Externalizing behaviors in adolescence often persist into adulthood [39], with youth displaying the highest levels being four to five times more likely to develop disruptive behaviors and emotional disorders [40]. Adolescent aggression is linked to personal, family, and academic challenges, including higher depressive symptoms, stress, lower self-esteem, and less family cohesion [41]. Antisocial adolescents are more prone to substance use, anxiety, and mood disorders, along with impaired social functioning in adulthood [42]. These impacts are more severe when externalizing behaviors start in childhood and extend beyond adolescence and increase the risk of psycho-social issues in adulthood [39,40,41,42].

Increased screen time among preschool boys regardless of the violent content correlates with proactive aggression, physical aggression, and delinquency. This may be because more screen time reduces opportunities for social interaction with peers, potentially leading to fewer conflicts and less aggressive behavior, as suggested by the time displacement hypothesis [4]. It might also be that exposure creates a mindset which predisposes affected individuals to resolve social competition, challenges, and conflict with unkind, pessimistic, and less tolerant behavior [1,2,3,37]. Studies by Lavados-Romo et al. (2021) [43] also suggest that higher screen time is associated with lower quality of life, including increased loneliness and weaker peer attachment. Previous research has found a prospective-longitudinal association between exposure to violent TV content and social withdrawal in the same participants from this study at ages 10 and 12, especially boys, suggesting that they also tend to isolate themselves socially [4,6].

There are theoretical explanations for these findings. Foremost are the established effects of social modelling at a crucial point in psycho-social development [14]. Exposure likely yields aggressive thoughts, feelings of anger, and arousal levels which are influenced by personal and situational factors, which in turn impact evaluation and decision-making processes to determine subsequent aggressive or non-aggressive behaviors [2]. A connection can be drawn between exposure to media violence and proactive and physical aggression in individuals [3]. Vicarious exposure at an age when children may not have strong cognitive capability of differentiating between fiction and reality can bias the perception of the preschoolers and influence how they react to it [14,15]. This leads the exposed youths to make more hostile attributions towards others and to act aggressively in ambiguous social situations, as they perceive the world as dangerous [43,44]. Such social-cognitive information processes may eventually become ingrained, contributing to explaining why children exposed to television violence exhibit more antisocial behaviors in adolescence [37].

We only found significant associations for boys. This is not surprising, given sex differences in preferences, where boys show more of an inclination toward fast-paced content of aggressive nature [27]. This is in line with social learning theory, which found that boys exhibited more physical aggression than girls in all conditions of the Bandura Bobo doll experiment [14]. This experiment was based on the premise that children identify with similar individuals. It is plausible that boys exhibit more aggressive behaviors because the violent characters depicted in movies are predominantly male [1,2,3,15]. As aggressive behaviors are rewarded in films, the child becomes motivated to replicate such behaviors while navigating socially, even in the absence of threat [43]. Finally, children exposed to violent television content become desensitized to witnessing aggressive acts and trivialize their consequences [15]. They become less sensitive to suffering in others and therefore less empathetic to their pain and may even develop positive attitudes towards violence [43,44]. This helps to establish a connection between proactive aggression and antisocial behavior resulting from exposure to violent television content during childhood.

This study is not without limitations. Firstly, this study is not experimental; thus, we cannot make causal inferences. However, the large range of covariates that represent individual and contextual factors can start to rule out competing explanations. Secondly, whether these data are representative of true child exposure to violent content in real life remains a challenge. That is, the operationalization of violent televiewing as a single-item measure may have introduced substantial subjectivity and recall bias. A more rigorous approach to measurement would have implemented direct observation, detailed media diaries, or objective coding of television programs. Our violence exposure data were likely skewed toward social desirability by parents, and it is likely that the adolescent data were affected by social desirability as well. Moreover, the confound-controlled analyses were meant to isolate the violence exposure predictor from pre-existing and baseline child and family factors. Remarkably, despite these methodological challenges and rigors, we found significant long-term risks of antisocial behavior and aggression. Finally, we did not examine the intermediate mechanisms between the predictor and outcome, which could have helped to enrich the study in establishing more precise links between variables, leading to a deeper understanding of the relationship between violent televiewing during preschool years and externalizing behavior in adolescence.

The chief strength of this study is that the data used in this study come from QLSCD, a large representative sample of the Quebec population, employing a prospective longitudinal design that allowed the tracking of a middle-class birth cohort for over 20 years. The natural exposure of children to violent television content has been recorded, addressing ethical concerns associated with this measure. Consequently, children born in the late 1990s are part of the last cohort to have experienced early childhood without all the technology available today. In this regard, our historical data provide a more accurate assessment of television exposure, which was the primary media at that time. This reduces the risk of confounding variables that could distort the findings, such as private viewing contexts and the portability of digital devices that diminish parental supervision, or the variety of media available today that can be used in a multitude of contexts, making its control more challenging. Finally, our sex-stratified approach to analyses faithfully demonstrates distinctions between girls and boys. The age of the children at which early exposure to violent television content is measured, namely during early childhood, as well as the ability to predict impacts into adolescence, fulfilled a great need in the literature, as outlined in the APA technical report [37].

The identified associations between preschool exposure to violent television content and subsequent hostile and antisocial behaviors beyond a decade underscore the importance of clinical research and policy interventions. Early identification and intervention for children exposed to violent content can be crucial in mitigating the long-term impacts on aggressive behaviors. Research should further explore nuanced aspects of this relationship, including specific violent content influences and potential protective factors. Using mixed methods and including potential moderators or mediators would help achieve a deeper understanding of the phenomenon and more accurately identify which children and contexts are most at risk of being impacted by violent media. Stricter policies should be implemented regarding the distribution of content intended for preschool children to ensure that the material watched by young audiences is appropriate [37]. Knowledge transfer strategies in public health remain essential for fostering healthier developmental trajectories for children and adolescents. For example, integrating media literacy programs within schools has the potential to sensitize and enable children and parents to critically navigate and comprehend the insidious consequences of viewed content [1,2,3,43]. Finally, preventive intervention campaigns that target parents and communities about the potential repercussions of preschool exposure can potentially promise more optimal long-term child development.

## Figures and Tables

**Table 1:** Descriptive statistics for predictor, outcome, and control variables in girls and boys.

	Girls			Boys		
	M (SD)	Categorical Variables (%)	Range	M (SD)	Categorical Variables (%)	Range
*Predictor* (3.5 years and 4.5 years)						
Violent televiewing						
0 = Never exposed		54.5			41.6	
1 = Rarely exposed		35.4			40.8	
2 = Sometimes exposed		8.7			14.6	
3 = Often exposed		1.3			3.0	
*Outcomes* (15 years)						
Proactive aggression	0.20 (0.52)		0–8	0.31 (0.55)		0–8
Reactive aggression	0.31 (0.62)		0–6	0.73 (0.87)		0–5
Physical aggression	2.69 (2.48)		0–32	3.54 (2.83)		0–32
Antisocial behavior	0.27 (0.58)		0–10	0.50 (0.81)		0–9
*Control variables*						
Difficult temperament (1.5 years)						
1 = beyond 1 SD above the M		15.8			16.5	
Neurocognitive skills (2.5 years)						
1 = less than 1 SD below the M		18.3			19.1	
Physical aggression exposure at home (3.5 years)						
1 = Some exposure		6.1			6.1	
Overall screen time (15 years)						
1 = beyond 1 SD above the M		13.0			10.3	
Baseline aggressive behavior (1.5 year)						
1 = beyond 1 SD above the M		16.1			13.7	
Maternal depressive symptoms (5 months)						
1 = beyond 1 SD above the M		13.7			14.9	
Maternal education (5 months)						
1 = did not finish high school		18.9			18.5	
Parental history of antisocial behavior (5 months)						
1 = beyond 1 SD above the M		15.6			16.4	
Hostile parenting (1.5 years)						
1 = beyond 1 SD above the M		4.4			6.0	
Family dysfunction (1.5 years)						
1 = beyond 1 SD above the M		16.8			14.4	
Family income (3.5 years)						
1 = insufficient		16.1			15.9	
Family configuration (15 years)						
1 = single parent		16.0			14.4	

Notes. M = mean; SD = standard deviation. Analyses corrected for attrition bias. Data were compiled from the final master file of the Quebec Longitudinal Study of Child Development (1998–2013), ©*Gouvernement du Québec*, *Institut de la Statistique du Québec*.

**Table 2:** Unstandardized regression coefficients (standard error) for the adjusted relationship between pre-existing and concurrent child and family confounding characteristics between ages 5 months and 15 years and violent televiewing at ages 3.5 and 4.5 years for girls and boys.

Confound Control Variables	*b* (SE)	
	Girls	Boys
	Violent Televiewing (3.5 and 4.5 years)	
Difficult temperament (1.5 years)	0.01 (0.06)	0.08 (0.07)
Neurocognitive skills (2.5 years)	0.09 (0.06)	0.02 (0.06)
Physical aggression exposure at home (3.5 years)	0.30 (0.10) **	0.33 (0.11) **
Overall screen time (15 years)	0.15 (0.07) *	−0.07 (0.08)
Maternal depressive symptoms (5 months)	0.10 (0.07)	0.02 (0.07)
Maternal education (5 months)	0.01 (0.06)	0.01 (0.07)
Parental history of antisocial behavior (5 months)	0.16 (0.06) **	0.22 (0.07) ***
Hostile parenting (1.5 years)	−0.15 (0.11)	−0.13 (0.11)
Family dysfunction (1.5 years)	0.06 (0.06)	0.03 (0.07)
Family income (3.5 years)	0.14 (0.07) *	0.06 (0.73)
Family configuration (15 years)	−0.06 (0.06)	0.06 (0.07)
Baseline aggressive behavior (1.5 years)	0.17 (0.06) **	0.18 (0.08) *
R^2^	0.058 ***	0.047 ***

Notes. * *p* < 0.05, ** *p* < 0.01, *** *p* < 0.001. Analyses corrected for attrition bias. Data were compiled from the final master file of the Quebec Longitudinal Study of Child Development (1998–2013), ©*Gouvernement du Québec*, *Institut de la Statistique du Québec*.

**Table 3:** Unstandardized regression coefficients (standard error) for the adjusted relationship between preschool exposure to violent televiewing and antisocial behavior at age 15 years for girls and boys.

	Predictor and Confound Controls	*b* (SE)			
		Proactive Aggression	Reactive Aggression	Physical Aggression	Antisocial Behavior
Girls	Violent televiewing (3.5 and 4.5 years)	−0.024 (0.024)	0.036 (0.028)	−0.049 (0.114)	0.009 (0.027)
	*Control variables*				
	Difficult temperament (1.5 years)	0.061 (0.047)	0.038 (0.055)	0.320 (0.219)	−0.048 (0.052)
	Neurocognitive skills (2.5 years)	0.070 (0.044)	0.008 (0.051)	0.011 (0.204)	0.037 (0.048)
	Physical aggression exposure at home (3.5 years)	0.180 (0.072) **	−0.008 (0.084)	0.354 (0.338)	0.222 (0.080) **
	Overall screen time (15 years)	0.017 (0.050)	0.098 (0.059)	0.685 (0.236) **	−0.027 (0.056)
	Maternal depressive symptoms (5 months)	0.007 (0.050)	0.038 (0.059)	0.097 (0.235)	−0.021 (0.056)
	Maternal education (5 months)	0.013 (0.046)	0.058 (0.053)	0.194 (0.213)	0.032 (0.050)
	Parental antisocial behavior (5 months)	0.131 (0.047) **	0.171 (0.055) **	0.816 (0.221) ***	0.168 (0.052) ***
	Hostile parenting (1.5 years)	−0.006 (0.083)	0.079 (0.097)	0.073 (0.390)	0.081 (0.092)
	Family dysfunction (1.5 years)	0.002 (0.046)	0.004 (0.053)	0.138 (0.214)	0.001 (0.051)
	Family income (3.5 years)	0.048 (0.050)	0.191 (0.058) ***	0.722 (0.232) **	0.090 (0.055)
	Family configuration (15 years)	−0.071 (0.046)	−0.135 (0.054) **	−0.543 (0.217) **	−0.025 (0.051)
	Baseline aggressive behavior (1.5 years)	−0.021 (0.048)	−0.018 (0.056)	−0.112 (0.223)	0.027 (0.053)
	R^2^	0.026 *	0.046 ***	0.054 ***	0.034 **
Boys	Violent televiewing (3.5 and 4.5 years)	0.045 (0.022) *	0.066 (0.035)	0.264 (0.114) *	0.076 (0.032) *
	*Control variables*				
	Difficult temperament (1.5 years)	−0.060 (0.049)	−0.026 (0.077)	−0.115 (0.248)	0.028 (0.070)
	Neurocognitive skills (2.5 years)	0.041 (0.045)	0.033 (0.071)	0.172 (0.228)	0.008 (0.065)
	Physical aggression exposure at home (3.5 years)	−0.054 (0.074)	−0.113 (0.117)	−0.332 (0.378)	−0.031 (0.107)
	Overall screen time (15 years)	−0.231 (0.058) ***	−0.129 (0.091)	−0.562 (0.295)	−0.302 (0.084) ***
	Maternal depressive symptoms (5 months)	0.053 (0.050)	0.179 (0.080) *	0.683 (0.257) **	0.105 (0.073)
	Maternal education (5 months)	−0.007 (0.048)	0.168 (0.076) *	0.282 (0.246)	0.014 (0.070)
	Parental antisocial behavior (5 months)	0.101 (0.048) *	0.185 (0.075) **	0.778 (0.243) ***	0.260 (0.069) ***
	Hostile parenting (1.5 years)	0.007 (0.074)	−0.060 (0.118)	−0.012 (0.380)	0.051 (0.108)
	Family dysfunction (1.5 years)	−0.034 (0.051)	−0.146 (0.080)	−0.605 (0.259) *	−0.117 (0.073)
	Family income (3.5 years)	0.093 (0.050)	0.171 (0.080) *	0.404 (0.258)	0.163 (0.073) *
	Family configuration (15 years)	−0.067 (0.050)	0.016 (0.079)	−0.060 (0.256)	−0.066 (0.072)
	Baseline aggressive behavior (1.5 years)	0.022 (0.053)	0.081 (0.084)	0.110 (0.271)	0.081 (0.077)
	R^2^	0.037 ***	0.041	0.043	0.054

Notes. * *p* < 0.05, ** *p* < 0.01, *** *p* < 0.001. Analyses corrected for attrition bias. Data were compiled from the final master file of the Quebec Longitudinal Study of Child Development (1998–2013), ©*Gouvernement du Québec*, *Institut de la Statistique du Québec*.

## Data Availability

Data supporting reported results can be found at *Étude longitudinale du développement des enfants du Québec* (ELDEQ).
